# Strategies for incorporating patient-reported outcomes in the care of people with chronic kidney disease (PRO kidney): a protocol for a realist synthesis

**DOI:** 10.1186/s13643-018-0911-6

**Published:** 2019-01-12

**Authors:** Kara Schick-Makaroff, Onouma Thummapol, Stephanie Thompson, Rachel Flynn, Mehri Karimi-Dehkordi, Scott Klarenbach, Richard Sawatzky, Joanne Greenhalgh

**Affiliations:** 1 Faculty of Nursing, University of Alberta, Edmonton Clinic Health Academy 11405-87 Ave, Edmonton, Alberta T6G 1C9 Canada; 2 Faculty of Medicine and Dentistry, Division of Nephrology, University of Alberta, 11-112 Clinical Science Building, 11350-83 Ave, Edmonton, AB T6G 2G3 Canada; 3 Telfer School of Management, University of Ottawa, 55 Laurier Avenue East, Ottawa, Ontario K1N 6N5 Canada; 4 School of Nursing, Trinity Western University, 7600 Glover Road, Langley, BC V2Y 1Y1 Canada; 5 Centre for Health Evaluation and Outcome Sciences, St. Paul’s Hospital, 588- 1081 Burrard Street, Vancouver, V6Z 1Y6 Canada; 6 Sahlgrenska Academy, University of Gothenburg, Medicinaregatan 3, Box 400, 405 30 Gothenburg, Sweden; 7 Sociology and Social Policy, University of Leeds, 11.21, Social Sciences Building, Leeds, LS2 9JT UK

**Keywords:** Clinical kidney practice, Patient-reported outcomes (PROs), Patient-reported outcome measures (PROMs), Patient-reported experience measures (PREMs), Person-centered care, Knowledge translation, Realist synthesis/review, Quality of life, Quality of care

## Abstract

**Background:**

Patient-reported outcomes and experience measures (jointly referred to here as PROs) are internationally recognized as a means for patients to provide information about their quality of life, symptoms, and experiences with care. Although increasingly recognized as key to improving the quality of healthcare at individual (e.g., patients, caregivers, and providers) and aggregate (e.g., government, policy/system-wide decision-making) levels, there are important knowledge gaps in our understanding of how PROs are, and can be, used across different settings, particularly in nephrology to enhance person-centered care. This knowledge is needed for developing strategies to guide optimal use of PROs in nephrology care. Currently, no strategies exist. The purpose of this review is to address this knowledge gap by answering the following realist question: How can PROs be used to enhance person-centered nephrology care, both at individual and aggregate levels?

**Methodology:**

Realist synthesis is an explanatory approach to data synthesis that aims to explain how context and mechanisms influence the outcome of an intervention. An initial program theory will be developed through the systematic search of the published literature in bibliographic databases (Ovid MEDLINE, Ovid Embase, EBSCOhost CINAHL, Web of Science, and Scopus) on existing theories explaining how PROs are used in healthcare settings. This initial program theory will then be tested and refined through the process of realist synthesis, using context-mechanism-outcome configurations. A kidney-specific program theory will then be created to address the utilization of PROs in nephrology across individual and aggregate levels to augment person-centered care. Searching will be iterative and refined as data is extracted and analyzed using a pilot-tested context + mechanism = outcome heuristic. Throughout, we will consult methodological experts, research team practitioners, and the Patient Advisory Committee to help refine the theories. Last, we will develop and disseminate knowledge translation products widely to knowledge user groups.

**Discussion:**

The utilization of PROs remains a challenge in nephrology. The findings from this synthesis will provide a framework to guide both policy makers and practitioners on how to enhance person-centered care through successful utilization of PROs across individual and aggregate levels in nephrology.

**Systematic review registration:**

PROSPERO CRD42017056063

**Electronic supplementary material:**

The online version of this article (10.1186/s13643-018-0911-6) contains supplementary material, which is available to authorized users.

## Background

The impact of chronic kidney disease (CKD) and end-stage kidney disease on the lives of those living with the illness is a vital, yet often neglected, outcome [1–4]. Internationally, there is emerging interest regarding the use and impact of patient-reported outcomes (PROMs) and patient-reported experience measures (PREMs), jointly referred to here as PROs. PROMs refer to self-report instruments used to obtain appraisals from healthcare recipients (patients and family caregivers) about outcomes relevant to their quality of life (e.g., well-being, overall health, symptoms, functional status and other aspects of psychological, social, and spiritual quality of life) [5]. PREMs refer to “questionnaires measuring the patients’ perceptions of their experience whilst receiving care” [6]. Currently, there is a knowledge gap in our understanding of how PROs are, and can be, optimally used in person-centered nephrology care. By person-centered, we mean an approach to care that focuses on “getting the know the person”, including the patient and family, by considering their “history, values, beliefs, priorities, preferences, current situation, future aspirations, and how they make sense of what is happening to them” [7]. This partnership is critical to ensuring high quality of care and, ultimately, improved health outcomes [8, 9]. The aim of our synthesis is to develop a tailored, kidney-specific program theory and subsequent knowledge translation products, regarding utilization of PROs to enhance person-centered care.

Quality of life has been identified as the health outcome that is highly valued by CKD patients, and most useful to clinicians in understanding burden of treatment, particularly in dialysis care [2, 10]. The routine use of PRO data in practice has been found to result in positive outcomes for patients, such as improved communication and enhanced care, and has revealed areas of concern that may otherwise have gone unnoticed [11–13]. However, findings from previous studies on PRO impacts have been mixed [13–15]. For example, the feedback of PRO scores is rarely a factor in clinicians’ decisions concerning treatment [13, 14] or has no impact on the process or outcomes of patient care [15].

Internationally, there has been heightened attention to the use of PROs in clinical practice with greater acceptance and recognition of the importance of patient-oriented approaches in nephrology [16, 17]. Interest in PRO use is increasing due to the recognition by regulators and practitioners of the necessity for patient-oriented approaches to kidney care [10, 16, 17]. In the USA, there has been an uptake of PROs because the Centers for Medicare and Medicaid Services has mandated routine assessment of quality of life for all end-stage kidney disease patients [18] as a prerequisite for coverage. However, most centers are not yet integrating this data into clinical practice to inform patient care [19, 20]. This neglected, untapped PRO data presents significant opportunity to not only bolster patient involvement in their own care, but also provide insights into patients’ needs and priorities at the point of care.

Despite the growing impetus to obtain PRO data and the opportunities it has to inform clinical care, there is uncertainty about how to best use it and fully integrate it into nephrology care at the individual and aggregate levels. Further, the nephrology community has identified its own deficiencies in providing person-centered care that fosters patient engagement for people living with advanced chronic or end-stage kidney disease [21]. In light of the trend towards person-centered care in nephrology, focused attention on person-centered strategies for utilization of PROs is both timely and needed. Our research will provide a novel approach, using realist synthesis, regarding PRO use as a complex health intervention in a kidney context to provide an explanatory analysis of what is necessary to ensure successful utilization of PROs to enhance person-centered care. In this paper, we present our realist synthesis protocol.

## Methods

### Aim

The aim of this study is to conduct a “realist synthesis” of strategies that will provide guidance on how PROs may be fully utilized in clinical nephrology care, both at individual (e.g., patients, family caregivers, and healthcare providers) and aggregate (e.g., government, policy or system-wide levels of healthcare) levels of decision-making. Realist synthesis, an established synthesis methodology with well-established guidelines [22], provides the means to not only review evidence on the complex intervention of PRO feedback at individual and aggregate levels of kidney healthcare, but also provide clarifying analysis of what is necessary to ensure successful utilization of PROs. The research question is as follows: How can PROs be used to enhance person-centered nephrology care, both at individual and aggregate levels? For the purpose of our study, clinical nephrology practice will include pre-dialysis care, dialysis (all modalities), pediatric kidney disease, and kidney transplantation, purposefully kept broad to facilitate theoretical applicability, recognizing then the need for tailoring to address the local context.

In alignment with realist synthesis, our objectives are:

1. To understand theories that explain how PROs are used
2. To develop a kidney-specific program theory about use of PROs in nephrology that may enhance person-centered care by testing and refining the theory through a realist synthesis of the empirical literature
3. To develop knowledge translation (KT) products for kidney practitioners and knowledge users that will facilitate the optimal utilization of PROs in nephrology care.

### Ethical considerations

This synthesis will not require ethical approval by the University of Alberta because all documents are in the public domain. The protocol is registered on the PROSPERO database (registration number: CRD42017056063) [23].

### Design: review methodology

Realist synthesis is an established theory-driven methodology for synthesizing knowledge from both qualitative and quantitative studies with the purpose of understanding *why* and *how* an outcome happens [22]. The emphasis is explanatory, focused on how the intervention brings about an outcome according to various contextual factors rather than a judgmental yes or no answer to the question, “does it work?” [24]. Outcomes are not defined a priori. Given that the use of PROs with kidney practitioners and patients, as well as within health organizations, is a complex intervention (e.g., logistical challenges in administration, changing practice patterns/behaviors, dependence on contextual factors), a realist synthesis is an appropriate approach. Realist synthesis requires the engagement of stakeholders to make the findings relevant in a way that goes beyond a “one size fits all” approach to problem-solving, thus generating transferable knowledge through heterogeneous views and across a range of settings [24].

In this review, we will seek to explain how the use of PROs in nephrology care as an intervention produces a chain of events that leads to both intended and unintended outcomes both at individual and aggregate levels. Using context-mechanism-outcome configurations, we will identify how contexts shape the mechanisms (triggered processes or behaviors, including the ways in which people respond to the resources offered by an intervention) through which the intervention (PRO feedback) brings about an outcome (e.g., enhanced patient engagement and activation). See Table 1 for definitions of context, mechanism, and outcome.

**Table 1:** Key definitions: context, mechanism, and outcome

Term	Definition
Context	The conditions “that triggers and/or modifies the behaviour of mechanisms” [41].
Mechanism	The causal forces that generate outcomes, yet they are not linear, arising from the diverse participants and contexts [22].
Outcome	The “intended outcomes (did the project succeed against the criteria it set itself at the outset)…, the intermediate outcomes as well as unplanned and/or unexpected impacts” [42].

An overview of the seven stages of this realist synthesis is outlined in Table 2 and described below. All stages below follow publication standards for realist syntheses [25] and follow Pawson’s realist methodology [24, 26]. In addition to these stages, we follow the PRISMA-P guidelines (see Additional file 1).

**Table 2:** Stages of realist synthesis

Stage of realist synthesis	Relationship to study objective	Current progress
1. Identify and refine scope and focus of review	Obj. 1: To understand theories that explain how PROs are used	Completed
2. Create an initial program theory to be tested and refined through evidence	Obj. 1	Completed
3.Search for evidence	Obj. 2: To develop a kidney-specific program theory about use of PROs in nephrology that may enhance person-centered care by testing and refining the theory through a realist synthesis of the empirical literature	Initiated
4. Screen, select, and appraise articles	Obj. 2	To be completed
5. Extract and code the data.	Obj. 2	To be completed
6. Synthesize extracted evidence and refine program theory	Obj. 2	To be completed
7. Develop and disseminate KT products	Obj. 3:To develop knowledge translation (KT) products for kidney practitioners and knowledge users that will facilitate the optimal utilization of PROs in nephrology care.	To be completed

#### 1. Identify and refine scope and focus of review

Based on findings from preliminary database searches and readings, the focus of our realist synthesis was refined. As the scope of the review was being defined, our international methodology expert (JG) facilitated a number of brief methodology workshops for the research team prior to the realist synthesis being conducted.

Engaging stakeholders is an integral part of the realist synthesis process to refine the scope of the review [27]. Thus, invitations for people living with kidney disease to participate in the Patient Advisory Committee were electronically circulated through the Kidney Foundation of Canada (KFOC), Northern Alberta and Territories Branch membership, as well as through local KFOC public presentations. Over a period of multiple months, 13 kidney patients (dialysis and transplant) and spouses volunteered. The primary investigator (KSM), a co-investigator (ST), and research assistants also met with the Patient Advisory Committee (a total of 15 people, including a patient Co-Chair) in May 2017 for a full-day training on practical strategies of how to engage in our patient-oriented research, sponsored by Strategies for Patient-Oriented Research [28]. The Patient Advisory Committee met again in August 2017 to give an update on the review process, to seek input on study objectives, and to discuss future development of knowledge translation materials that they would find useful.

#### 2. Create an initial program theory to be tested and refined through evidence

Searching in realist synthesis comprises two main stages: (1) searches to identify existing implementation theories and frameworks and (2) searches for evidence to test theories relevant to the review questions [24]. From data from the first search, we will identify an initial program theory (or theories) that explains how an intervention is intended to work. The initial program theory will encompass context-mechanism-outcome hypotheses to provide a theoretical explanation about how PROs are used at individual and aggregate levels across healthcare practices.

**Fig. 1:**
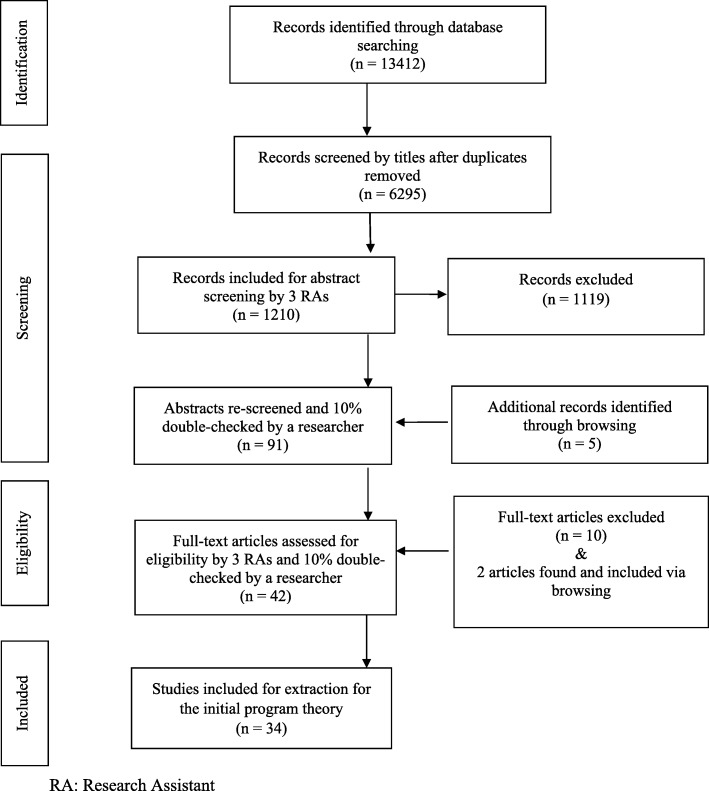
Screening process for the initial program theory

To locate these theories, we (a) systematically searched the literature to identify existing theories and (b) consulted our methodological experts and research team practitioners. Research team members identified three exemplar papers a priori [29–31]. A research librarian was consulted to design search strategies. A preliminary search strategy was developed for Ovid MEDLINE [32]. It was further expanded to focus on published literature, indexed in the following bibliographic databases: Ovid MEDLINE, Ovid Embase, EBSCOhost CINAHL, Web of Science, and Scopus. The search was conducted on January 20, 2017. The search contained two main concepts: patient reported outcome measures and theory. Appropriate subject headings and keywords were used in the search and were modified for each specific database, in order to retrieve literature about each of these concept areas [32]. The search was not limited by language, type of literature (primary research, theoretical, or review) or date to ensure breadth of scope. In total 13,412 articles were retrieved. Of these, 7117 were duplicates (1516 were automatically de-duplicated in Ovid and 5601 were de-duplicated using endnote). An endnote library containing 6295 records was provided to team for screening. Titles were screened and 1210 abstracts proceeded for screening by the team (with 10% double-screened for relevance). Inclusion and exclusion criteria for objective 1 are outlined in Table 3. Rater agreement between reviewers was 91.7% (Table 4). Thirty-four full-text articles were included for extraction for the initial program theory (Fig. 1).

**Table 3:** Inclusion/exclusion criteria for objective 1 (to understand theories that explain how PROs are used)

Inclusion	Exclusion
(Must include 1 below and must include either 2 or 3 or 4 or 5)	
1. All PRO measures (e.g., healthcare provider outcomes, quality of care outcomes) either at individual (patients, healthcare providers) OR aggregate (system e.g., use of the PRO information for quality improvement purposes) levels, OR healthcare experience measures. 2. The main focus of the study is to include a formal or substantive theory, mid-range theory, theoretical/conceptual framework, or model on the use of PROs that describes how individual/aggregate PROs are intended to work. 3. The main focus of the study is to review/provide ideas about how individual/aggregate PRO use is intended to work or provides a critique of the ideas underlying how individual/aggregate PRO use is intended to work. 4. The main focus of the study is to provide stakeholder accounts or opinions of how individual/aggregate PRO use does OR does not work. 5. The main focus of the study is to outline, discuss, or review potential unintended consequences of individual/aggregate PRO use.	1. Articles not written in English. 2. The main focus of the paper is to report findings in which a PRO is used as a research tool [e.g., an evaluation of an intervention, a study exploring the health-related quality of life of specific populations, a study focuses mainly on the statistical analysis]. 3. The main focus of the paper is to focus on evaluating the psychometric properties of a PRO or on reviewing the psychometric properties of a PRO or a collection of PROs. 4. The paper’s main focus is to provide advice or recommendations about which PRO to use in a research context.

During screening, the following criteria were applied. We asked the question, “Does this provide theoretical explanation about how PROs are used at an individual or aggregate level?”

**Table 4:** Rater agreement

No difference between the raters	Number of items
121	132
Percent agreement	91.7%

In order to assess rater agreement between two groups of raters, we first compiled the primary results (rated by the research coordinator and research assistants) into one group, and all the 10% double-screening results into another group. The percentage agreement between the two groups was 91.7%. Agreement between raters can be related to the initial practice screening at the very early stage, and the frequent team meetings that were regularly conducted to discuss all uncertainties that accumulated throughout the screening process

An initial program theory will be built to map out the main components of using PROs, anticipated outcomes, elements that contribute to these outcomes, and sequencing of these elements. The initial program theory (or theories) will be presented to various stakeholders (e.g., interdisciplinary nephrology practitioners). We will ask them to identify and discuss which context-mechanism-outcome hypotheses are most important. After this meeting, the initial program theory will be refined prior to synthesis of the renal literature [22].

The following four stages address objective 2: To develop a kidney-specific program theory about use of PROs in nephrology that may enhance person-centered care by testing and refining the theory through a realist synthesis of the empirical (renal) literature.

#### 3. Search for evidence

The purpose of the second search is to identify relevant bodies of literature as “data” from which to test and refine our initial program theory for a kidney context. The search strategy will be guided by the initial program theory/theories, and it will examine the relevant bodies of literature pertaining to theoretical use of PROs, as well as observed quantitative and qualitative research on the use of PROs in clinical nephrology care at individual and aggregate levels.

In collaboration with the library scientist on the research team, we have developed an initial database search strategy for Ovid MEDLINE [32]. The search strategy was reviewed by PRESS (Peer Review of Electronic Search Strategies). Searches in other databases (Ovid Embase, EBSCOhost CINAHL, Web of Science, and Scopus) will be based upon this search strategy using appropriate subject headings and operators for each database. The search will not be limited by language, type of literature (primary research, theoretical, or review), or date to ensure breadth of scope.

We will also search the gray literature, and consider white papers, editorials, reports, and guidelines describing the use of PROs in clinical nephrology practice. This will be done by searching in the bibliographic databases that index gray literature, using web-based search engines such as Google and DuckDuckGo, clinical practice guideline indices, and looking at the websites of organizations involved in kidney healthcare such as the Kidney Foundation of Canada and the National Kidney Foundation. The practitioners on the research team will help refine search terms to ensure that they are sufficient to capture the broad manner in which PROs are utilized in different practice environments and healthcare systems.

As the search results are screened, additional key articles will be identified. Through this iterative process, we will use forward citation chaining (articles that cite a given article) and backward citation chaining (searching the reference list of a given article) [33] using Google Scholar, and in-depth exploration of the relevant studies’ relationship to other research articles. Searching for additional data to enhance development of the program theory is a critical step in realist synthesis. We anticipate that additional searches will be undertaken to test and develop specific sections of the program theory. Thus, the research team will refine the screening processes based on future searches.

#### 4. Screen, select, and appraise articles

We have developed the inclusion and exclusion criteria (for the second objective) for abstract screening (see Table 5). In review of this literature, we will ask the question, “Does this provide any evidence, discussion, or conceptual/theoretical perspectives that will enable us to test and refine our program theory for PRO use in clinical nephrology care to enhance person-centered care?”

**Table 5:** Inclusion/exclusion criteria for objective 2 (to develop a kidney-specific program theory about use of PROs in nephrology that may enhance person-centered care by testing and refining the theory through a realist synthesis of the empirical literature)

Inclusion	Exclusion
(Must include 1, 2, and 3 and must include 4 or 5 or 6)	(Must exclude at least one of 1 or 2 or 3)
1. Source focused on nephrology population (e.g., patients [adults, pediatrics, transplant, CKD patients of all forms] and/or practitioners). 2. Source types: All study designs and other discourses, such as theoretical discussions, literature reviews, editorials, or guidelines, surrounding use of PROs in clinical nephrology practice. 3. Source written in English. 4. Use of PROs in individual clinical nephrology practice. 5. Evidence on the use of aggregate PRO information for micro-, meso-, or macro-levels in nephrology. 6. Involve a formal or substantive theory, mid-range theory, theoretical/conceptual framework, or model on PROs use in clinical nephrology practice or for healthcare administration purposes.	1. The source is focused on exploring, evaluating, or reviewing psychometric properties of PRO use in nephrology (e.g., validity, reliability). 2. The source is focused on development of new PRO. 3. The source is focused on reporting findings in which a PRO is used as a research tool (e.g., an evaluation of an intervention, a study exploring the health-related quality of life of specific populations).

In review of this literature, we will ask the question, “Does this provide any evidence, discussion, or conceptual/theoretical perspectives to test and refine our initial program theories for PRO use to enhance person-centered nephrology care at individual and/or aggregate levels?”

The selection of the articles will be based on inclusion/exclusion criteria, relevance to theory building and/or testing, and rigor [25]. After all abstracts are screened, the research coordinator and research assistants will screen full-texts. To attend to rigor, 10% double-screening and discussion within the research team will be undertaken to discuss any discrepancies. Study quality will be judged according to quality standards appropriate for the type of research and on relevance (whether the article contributed to theory building). The quality will be assessed using McGill Mixed Methods Appraisal Tool (MMAT), a critical appraisal tool developed for appraising the methodological quality of studies included in systematic reviews [34]. Unlike many appraisal tools, the MMAT has documented evidence of reliability [34]. Studies will not be excluded based on MMAT scores because relevance will be based on contribution to context-mechanism-outcome configurations and provision of relevant evidence to help test the theory/theories.

Appraisal continues as evidence is extracted for its relevance to theory testing and the rigor with which it has been produced [35]. In many instances, it is only a subset of findings from each study that relate specifically to the theory being tested that are included in the synthesis. Therefore, quality appraisal relates specifically to the validity of the causal claims made in this subset of findings, rather than the study as a whole. Trust in these causal claims is also enhanced by the accumulation of evidence from a number of different studies which provide further lateral support for the theory being tested, discussed in more detail below.

#### 5. Extract and code the data

Given that any section of a document may be pertinent to the theory enhancement, detailed data management processes are essential. NVIVO, a qualitative software system may be used to create a filing system and coding database. The research coordinator and research assistants will complete data extraction. To ensure consistency, a random selection of articles will be independently coded by the principal applicant (KSM), research team members (ST, RF), the research coordinator and assistants. KSM will join as a reviewer to resolve differences. The coding will be deductive (codes created in advance of data extraction and analysis based on the initial program theory) and inductive (codes created to capture data reported in the included studies). Both qualitative and quantitative data are compiled. In addition, the inferences and conclusions drawn by the authors of the studies are extracted as data within realist synthesis, as they often permit the identification of sub-theories which can then be further tested with empirical evidence. Different fragments of evidence are sought and utilized from each study. Data will also be coded to identify if it pertains to context, mechanism, or outcome in order to explain and understand how, and in what circumstances, PROs enhance person-centered nephrology care at individual and aggregate levels.

#### 6. Synthesize extracted evidence and refine program theory

Synthesis of the extracted evidence will focus on testing and refinement of the initial program theory to create a kidney-specific theory about use of PROs at individual and aggregate levels in nephrology care. Data will be synthesized across the literature into context-mechanism-outcome configurations seeking to explain how PROs are used to enhance person-centered nephrology care at individual and aggregate levels. For example, for the purposes of understanding how context influences outcomes, we will compare use of PROs in nephrology practice where it was “successful” against those which were not, or where PROs were completed or implemented electronically or through an electronic medical record versus paper. Guided by the literature, the research team will then identify which mechanisms are “key” in this process.

As extracted evidence is synthesized, the initial program theory/theories will be refined to reflect evidence from the renal literature. During this process, we will ask the following questions: “What does this evidence suggest about this aspect of our theory? Does it support it? Does it disprove it? Does it suggest an amendment to it?” [22]. The initial program theory/theories containing context-mechanism-outcome configurations will then be amended in response to these questions. New context-mechanism-outcome configurations may be created, some initially hypothesized configurations may be removed, and others may be combined, separated, or revised.

These refinements include both summary and analysis, but do not yet offer synthesis in its fullest sense. Moving up a level of abstraction to “make sense” of the patterns of findings involves using a priori middle-range theories or formal theories from the field in which the analysis may be situated [22]. Examples may include theories of change, learning theories, systems theories, or complexity theories. With the addition of formal theories, syntheses may bridge the evidence provided in the renal literature with theoretical “sense-making” of the patterns.

Although the processes of searching, screening, selecting, extracting, and synthesizing have been described in a linear fashion, the review process is iterative. During the analytic steps, we will move iteratively between analysis of examples in the data, revision of the program theory, and refined searching of the data to test specific sections of the theory. To attend to rigor, refinement of the theory will include consultation with research team nephrology practitioners and the Patient Advisory Committee. Testing and refinement of the theory is the purpose of the synthesis itself, rather than “validation” per se. The overarching purpose is to build an explanation of how context shapes the mechanisms through which the intervention works. The emerging explanation will be substantiated in evidence, and stakeholder involvement throughout the synthesis will support the usefulness of the emerging explanation. However, it would be beyond the scope of the synthesis to directly test its usefulness. The next and last stage addresses objective 3: To develop KT products for kidney practitioners and knowledge users that will facilitate the optimal utilization of PROs in nephrology care.

#### 7. Develop and disseminate KT products

Our integrated KT products will (1) provide evidence-based strategies for PRO use in clinical nephrology practice that enhances person-centered care and (2) provide guidance to policy makers on effective, strategic processes for PRO use in kidney settings. The development of KT products will be created with input from the Patient Advisory Committee and CIHI’s Patient-Reported Outcome Measures Renal Care Working Group.

The KT products from this project will be circulated and disseminated widely from the team to knowledge user groups such as CIHI’s Patient-Reported Outcome Measures Renal Care Working Group; Canadian Association of Nephrology Nurses and Technologists; Canadian Association of Nephrology Social Workers; Canadian Society of Nephrology, as well as through publications and conference presentations. Our intention in the translation of our findings into knowledge products is that the results may be both meaningful and useful for practitioners and policy makers integrating PROs in renal settings.

Throughout the course of the project, our team will work towards creation of the following products:

1. A theoretical framework to guide PRO use across a range of kidney settings;
2. Recommendations for knowledge users on effective strategies that may be applied to program and policy development;
3. Recommendations on how to tailor the intervention to local circumstances; and
4. Training materials to support the use of PROs in nephrology care, such as webinars, a handbook, or a “how to” publication with practical advice on using PROs.

These products will be disseminated widely through our knowledge users in their own areas of influence, as well through publications and conference presentations.

## Discussion

While the use and integration of PROs has been broadly addressed in clinical practice, challenges pertaining to the implementation of PROs in nephrology remain, both at individual and aggregate levels. While some synthesis work has been previously undertaken regarding PRO use [36–38], no comprehensive synthesis has focused on the kidney context. Given the high symptom burden and low quality of life reported by people with kidney disease, this is an important knowledge gap to address. Thus, there is scarce information for practitioners and policy makers caring for kidney patients. The findings from this realist synthesis will provide a theoretical framework to guide both policy makers and practitioners on how to enhance person-centred care through successful utilization of PROs across individual and aggregate levels in nephrology.

Although our protocol is comprehensively developed following well-established guidelines for realist synthesis [22, 25], there are limitations that need to be taken into account. First, our review is limited to the English language. As a result, cultural variability in evidence from studies conducted in countries where English is not the dominant language may not be adequately represented. Second, the protocol emphasizes reliance on published sources. While other search strategies will be included, published sources will likely produce the highest yields, thus results may be influence by the well-documented concern of publication bias.

PROs are increasingly used to inform higher levels of healthcare administration and decision-making. For example, CIHI, in collaboration with the Canadian Organ Replacement Register, is addressing how PRO use in renal care may become standardized data collection. Both the USA and UK have already moved towards consistent PRO reporting. Yet integrations of such reports for use in practice or administrative levels have not been widely assessed. This realist synthesis project will provide timely evidence needed for practitioners and policy makers to better target complex interventions to local circumstances.

There has been increasing calls for a useful practical approach to knowledge synthesis that responds to relevant information needs of practitioners and policy makers to make informed decisions about practices and policies, and ensure implementations, particularly in relation to PROs use [27, 39]. In complex health and social interventions, a traditional knowledge synthesis approach such as systematic reviews is not well suited given its inability to explain heterogeneous results and lack of attention to context [40]. Realist synthesis is a suitable approach to reviewing research evidence on complex interventions in a renal context, which provides an explanatory analysis of what is necessary to ensure successful utilization of PROs in order to enhance person-centered care.

This realist synthesis adopts an integrated knowledge translation approach using meaningful engagement and significant collaboration among researchers, knowledge users, and Patient Advisors throughout the review process. This approach will be undertaken to ensure that the outputs are relevant and useful to knowledge users, and may ultimately lead to improved patient health outcomes. The results of this review will be of interest to policy makers and administrators considering where and how to allocate resources so that PROs are optimally utilized. Findings from this realist synthesis will offer strategies to guide use of PROs to enhance person-centered nephrology care.

## Conclusion

Despite international attention to use of PROs in clinical care and recognition of the importance of patient-oriented approaches, the routine utilization of PROs remains a challenge in nephrology. Our review will address this knowledge gap and develop a tailored, kidney-specific program theory, and subsequent knowledge translation products, that optimize use of PROs across individual and aggregate levels of person-centered care in nephrology.

## Additional file

Additional file 1: PRISMA-P 2015 Checklist (DOCX 33 kb)

## Abbreviations

CIHI: Canadian Institute for Health Information
CKD: Chronic kidney disease
KT: Knowledge Translation
PREMs: Patient-Reported Experience Measures
PRESS: Peer Review of Electronic Search Strategies
PROMs: Patient-Reported Outcomes Measures
PROs: Patient-Reported Outcomes and Experience Measures
